# CRISPR/Cas9-Mediated Targeted Mutagenesis of *GmUGT* Enhanced Soybean Resistance Against Leaf-Chewing Insects Through Flavonoids Biosynthesis

**DOI:** 10.3389/fpls.2022.802716

**Published:** 2022-02-22

**Authors:** Yongxing Zhang, Wei Guo, Limiao Chen, Xinjie Shen, Hongli Yang, Yisheng Fang, Wenqi Ouyang, Sihua Mai, Haifeng Chen, Shuilian Chen, Qingnan Hao, Songli Yuan, Chanjuan Zhang, Yi Huang, Zhihui Shan, Zhonglu Yang, Dezhen Qiu, Xinan Zhou, Dong Cao, Xia Li, Yongqing Jiao

**Affiliations:** ^1^Key Laboratory of Biology and Genetic Improvement of Oil Crops, Ministry of Agriculture and Rural Affairs, Oil Crops Research Institute, Chinese Academy of Agricultural Sciences, Wuhan, China; ^2^National Key Laboratory of Crop Genetic Improvement, College of Plant Science and Technology, Huazhong Agricultural University, Wuhan, China; ^3^Collaborative Innovation Center of Henan Grain Crops, College of Agronomy, Henan Agricultural University, Zhengzhou, China

**Keywords:** CRISPR/Cas9, soybean, UDP-glycosyltransferase, *Helicoverpa armigera* Hübner, *Spodoptera litura* Fabricius

## Abstract

Leaf-chewing insects are important pests that cause yield loss and reduce seed quality in soybeans (*Glycine max*). Breeding soybean varieties that are resistant to leaf-chewing insects can minimize the need for insecticide use and reduce yield loss. The marker gene for QTL-M, *Glyma.07g110300* (LOC100775351) that encodes a UDP-glycosyltransferase (UGT) is the major determinant of resistance against leaf-chewing insects in soybean; it exhibits a loss of function in insect-resistant soybean germplasms. In this study, *Agrobacterium*-mediated transformation introduced the CRISPR/Cas9 expression vector into the soybean cultivar Tianlong No. 1 to generate *Glyma.07g110300-*gene mutants. We obtained two novel types of mutations, a 33-bp deletion and a single-bp insertion in the *GmUGT* coding region, which resulted in an enhanced resistance to *Helicoverpa armigera* and *Spodoptera litura*. Additionally, overexpressing *GmUGT* produced soybean varieties that were more sensitive to *H*. *armigera* and *S. litura*. Both mutant and overexpressing lines exhibited no obvious phenotypic changes. The difference in metabolites and gene expression suggested that *GmUGT* is involved in imparting resistance to leaf-chewing insects by altering the flavonoid content and expression patterns of genes related to flavonoid biosynthesis and defense. Furthermore, ectopic expression of the *GmUGT* gene in the *ugt72b1* mutant of *Arabidopsis* substantially rescued the phenotype of *H. armigera* resistance in the *atugt72b1* mutant. Our study presents a strategy for increasing resistance against leaf-chewing insects in soybean through CRISPR/Cas9-mediated targeted mutagenesis of the *UGT* genes.

## Introduction

Soybean (*Glycine max*) is an important industrial crop that provides edible oil, vegetable protein, and active compounds, such as flavonoids (Arifin et al., 2021). Leaf-chewing insects, such as *Helicoverpa zea*, *Helicoverpa armigera* Hübner, and *Spodoptera litura* Fabricius, are important insect pests that cause a loss in yield and a decline in the quality of soybeans (Otuka et al., 2020; Haile et al., 2021). Farmers mainly depend on chemical pesticides to control this threat. However, the long-term use of chemical pesticides leads to the development of drug resistance in the target insects, contaminates land and water resources, and increases costs of environmental management (Elba et al., 2014; Bondori et al., 2018). Breeding soybean varieties that are resistant to leaf-chewing insects can minimize the need for insecticides, improve soybean yields, and reduce concerns stemming from pesticide use (Ortega et al., 2016).

Transgenic breeding offers key breakthroughs in plant breeding, and the cultivation of transgenic crops has appreciably increased world agricultural productivity in the past two decades (Raman, 2017). Genetically modified cultivars expressing insecticidal proteins have become vital to the effective management of leaf-chewing insect pests. Major insecticidal proteins include the crystal protein (CRY), the vegetative insecticidal protein (VIP), and the protease inhibitor (PI), whose genes have been used to develop insect-resistant genetically modified cultivars (Saikhedkar et al., 2019; Kumar et al., 2020). Many insect-resistant transgenic crops now exist; there are six commercialized soybean cultivars with various insect-resistance (IR) genes (Ichim, 2019; Kumar et al., 2020). This genetic trait provides an effective control strategy for defoliators, including *H. armigera*. However, transgenic crops that have foreign DNA randomly integrated into their genomes have always been controversial, owing to the durability of resistance, environmental safety, and potential adverse health effects on consumers, limiting such transgenic breeding (Domingo and Bordonaba, 2011; Raman, 2017; Schiemann et al., 2019; Pozebon et al., 2020; Gao, 2021).

In addition to the deployment of genetically modified soybean varieties that overexpress one or more insecticidal protein genes, other soybean varieties have been screened for native resistance to leaf-chewing insects, such as *H*. *armigera* (Haile et al., 2021). Zhu et al. (2006) fine-mapped a major quantitative trait locus (QTL-M) for insect resistance in PI 229358 and developed Benning near-isogenic lines (NILs) resistant to *H. zea.* Similarly, several studies have reported insect-resistance QTLs against *S. litura* (Kim et al., 2013, 2015; Liu et al., 2016). Ghione et al. (2021) found four markers associated with resistance genes against stink bugs using the association mapping strategy. To date, several insect-resistance QTLs, specific for leaf-chewing insects, have been identified in the soybean germplasm (Rector et al., 2000; Zhu et al., 2008; Wang et al., 2015; Ortega et al., 2016; Sabljic et al., 2020; Ghione et al., 2021; Lucini et al., 2021). Interestingly, Ortega et al. (2017) reported that the *Glyma.07g110300* marker (encoding a UDP-glycosyltransferase) in QTL-M was a functional single-nucleotide polymorphism (SNP) that could be used for the marker-assisted selection of insect-resistance QTLs and *Glyma.07g110300* exhibits a loss of function in insect-resistant soybean germplasms. Native resistance traits have been reported against *H. zea*, but information about the *GmUGT* gene and its roles in resistance to *H. armigera* remains unclear.

The CRISPR/Cas9 (clustered regularly interspaced short palindromic repeat-associated endonucleases) is an efficient tool for genetic manipulation; it uses simple RNA-guided DNA recognition and binding for sequence-specific nucleic acid cleavage. The CRISPR/Cas9-based genome-editing can help produce crop varieties, similar to non-transgenic crops, without adding any foreign DNA to the genome, and thus, has become a powerful tool to advance crop breeding (Knott and Jennifer, 2018; Chen et al., 2019; Schiemann et al., 2019; Gao, 2021). This technology has been widely applied in soybeans (Bao et al., 2019; Cheng et al., 2019; Han et al., 2019; Cai et al., 2020). Therefore, CRISPR/Cas9-mediated construction of insect-resistant germplasms to reduce the dependence on pesticides has the potential to become a simple and feasible breeding method for soybeans in the future.

In this study, we employed the CRISPR/Cas9 system to create *GmUGT-*gene knockout mutants and analyzed the change in resistance of the mutant to leaf-chewing insects. The *H. armigera* and *S. litura* larval feeding assay demonstrated that the mutagenesis of *GmUGT*-improved soybean resistance against leaf-chewing insects by altering the biosynthesis pathway of flavonoids in soybeans. Furthermore, the ectopic expression of the *GmUGT* gene substantially rescued the phenotype of resistance against *H. armigera* in the *atugt72b1* mutant. Findings from this study present a strategy to increase the resistance to leaf-chewing insects in soybean and other crops through CRISPR/Cas9-mediated targeted mutagenesis of *UGT* genes.

## Materials and Methods

### Plant Materials and Growth Conditions

Soybean cultivar Tianlong No. 1 (WT), as well as transgenic varieties generated by the CRISPR/Cas9-mediated mutagenesis of *GmUGT* gene and *GmUGT*-overexpression, were grown in pots (height × top diameter = 18.5 cm × 18.5 cm). Three plants per pot were grown in an artificial climate chamber under 14-h light/10-h dark photoperiod conditions at 26°C. *Arabidopsis thaliana* Columbia-0 (Col-0) and the T-DNA insertion mutant, *atugt72b1*, were obtained from the Arabidopsis Biological Resource Center (ABRC). The Col-0, *atugt72b1*, and transgenic *Arabidopsis* lines were grown in pots (height × top diameter = 10 × 8 cm) with four plants per pot in an artificial climate chamber under 8-h light/16-h dark photoperiod conditions at 22°C.

### Vector Constructs and Plants Transformation

To edit the *GmUGT* gene, the pSC1-CRISPR/Cas9P_*GmUbi*3_-BK plasmids were constructed, following the method of Du et al. (2016). Briefly, the target adaptor was designed using the CRISPR-P^1^ web tool; it was then integrated into the single guide RNA (sgRNA) expression cassettes driven by the *GmU6* promoter, which was subsequently built into the pSC1-CRISPR/Cas9PGmUbi3-BK vector. The full-length coding sequence (CDS) of *GmUGT* was cloned from the Tianlong No. 1 complementary DNA (cDNA) library. The *GmUGT* CDS was subcloned into the pB2GW7.0 binary vector (Shen et al., 2018) through the LR recombinase reaction (Invitrogen, Carlsbad, CA, United States). The constructs, *P35S*-*GmUGT*-pB2GW7.0 and *PGmU6-sgRNA*-pSC1-CRISPR/Cas9P_*GmUbi*3_-BK, were introduced into *Agrobacterium tumefaciens* EHA105 by electroporation and then transformed into the WT as previously described (Chen et al., 2021).

For *Arabidopsis* transformation, the *GmUGT* gene was subcloned into pFGC5941 (Lei et al., 2021) driven by the *ATUGT72B1* promoter using the ClonExpress II One Step Cloning Kit (Vazyme, Nanjing, China); the clone, thus, prepared was introduced into the *Arabidopsis atugt72b1* mutant with EHA105 by the floral dip method (Clough and Bent, 1998).

### Identification of Transformants and Mutations

For PCR, genomic DNA was extracted from the leaves of the WT plants and the transgenic varieties generated by the CRISPR/Cas9-mediated mutagenesis of *GmUGT* and *GmUGT*-overexpression. Transgenic plants were tested with a Transgenic Plant *Bar* Gene Rapid Detection Kit (Institute of Oil Crops, Chinese Academy of Agricultural Sciences, Wuhan, China).

The PCR products were separated by electrophoresis on 1% *agarose* in 1 × Tris-acetate-EDTA (TAE) buffer to screen the *GmUGT* mutants. The purified DNA fragments with *Bar* gene detection primers were sequenced and analyzed. To identify mutations in the regenerated plants, amplified DNA surrounding the target regions of the sgRNA was sequenced and analyzed. The successfully edited types were identified by sequence alignment with the WT DNA sequence. The PCR identified *GmUGT*-overexpressing transgenic soybean plants with vector detection primers. The real-time quantitative PCR (qRT-PCR) was used to determine the expression levels of *GmUGT* in overexpressing transgenic lines. The *GmSKIP16* gene was used as the internal control in qRT-PCR analysis. Rescued lines of *Arabidopsis* were selected on the soil by spraying Basta at 1:1000 concentrations (Bayer CropScience, Monheim, Germany). The qRT-PCR was used to determine the expression levels of *GmUGT* in transgenic *Arabidopsis* lines. The *AtACTIN2* gene was used as the internal control in qRT-PCR analysis.

### Detection of Off-Target Sites

To consider the off-target effects, the potential off-target sites were predicted by the website CCtop^2^. The overlapping sequence between the similar position of the off-target site gene (*Glyma.09G245300*) and the sgRNA was 16-bp long. This gene was checked by PCR amplification and sequencing. Off-target site detection primers are listed in Supplementary Table 1.

### Analysis of Insect Resistance in Mutant and Transgenic Lines

For the larval feeding assay, soybean plants were grown until they had five fully unfolded leaves. *Arabidopsis* plants were grown until they had 10 fully unfolded leaves. The *H. armigera* and *S. litura* larvae obtained from Huazhong Agricultural University were hatched at 28°C from a single egg mass. To analyze insect resistance in the WT plant and the transgenic varieties from CRISPR/Cas9-mediated mutagenesis of the *GmUGT* gene and *GmUGT* overexpression, leaves were collected from the same positions. Four small rounded blades (each with a diameter of 4 cm) were collected from each leaf with a hole puncher. The leaves were placed in a Petri dish (90 × 15 mm) containing two pieces of soaked filter paper. A second instar larva was placed on each leaf. Three days after the treatment, representative photos were taken, and the leaf area of soybean varieties was measured using a leaf area meter. After feeding for 7 days, the weight per larvae was recorded. Two knockout mutants (*ko-3* and *ko-5*), three overexpressing transgenic lines (OX-1, OX-2, and OX-44), and the WT plants underwent fifty biological replicates. For the analysis of insect resistance in *Arabidopsis*, two whole plants were used as one sample. Plants were wrapped in moistened absorbent cotton and placed in 500 mL round disposable plastic boxes with 100 freshly hatched larvae. Three days after treatment, representative photos were taken. After feeding for 7 days, the weight per larvae and the number of surviving larvae were recorded. Three transgenic lines of soybean (RE-1, RE-2, and RE-3), *atugt72b1*, and Col-0 were tested in three biological replicates.

### Expression Pattern Analysis of *GmUGT*

The tissues of the WT plants were tested at different stages to analyze the expression pattern of *GmUGT* in soybean. Cotyledons, roots, simple leaves, trifoliate leaves, and stems were harvested from the WT at the V2 stage, flowers were harvested at the R2 stage, and pods at the R5 stage for RNA purification. The harvested samples were stored at −80°C until total RNA extraction was performed for gene expression analyses.

For the induced expression pattern analysis, 10 *H. armigera* larvae were placed on the first and second trifoliate leaves of the WT at the V4 stage. The leaves of the control and treated plants were excised at nine sampling times (0, 1, 2, 3, 6, 12, 24, 36, 48, or 72 h) after being attacked by *H. armigera* to identify the induced resistance. For mechanical damage treatment, the damage was simulated using a needle with 48 spines per leaf. A mix of damaged and undamaged leaves was collected. The *H. armigera* attack and mechanical damage treatments were performed in biological triplicates.

### RNA Isolation and Real-Time Quantitative PCR Analysis

Total RNA was extracted from plant tissues using TRIzol reagent (Invitrogen, Carlsbad, CA, United States). The RNA concentration was measured using NanoDrop One (Thermo Scientific, Waltham, MA, United States) for qRT-PCR and RNA sequence (RNA-seq) assays. One microgram of total RNA per sample was used to synthesize the first-strand of cDNA using the TransScript^®^ First-Strain cDNA Synthesis SuperMix according to the manufacturer’s instructions (TransGen Biotech, Beijing, China). A total volume of 20 μL was set up for the qRT-PCR that contained 0.5 μM forward and reverse primers, 10 μL of the SYBR premix, and 20 ng of the cDNA. The assay was performed in a QuantStudio™ 5 Real-Time PCR Instrument (Thermo Scientific, Waltham, MA, United States) under the following cycle conditions: 95°C for 30 s, 40 cycles of denaturation at 95°C for 5 s, annealing at 60°C for 30 s, and extension at 72°C for 30 s with simultaneous fluorescence measurement and a melting curve at 55–95°C (0.5°C increments with each cycle). Three biological replicates were assayed. The *GmSKIP16* (*Glyma.12g05510*), *AtACTIN2* (*At3G18780*), and *HaActin* (*GU182917*) were used as endogenous controls for experiments involving nucleic acids from *Arabidopsis*, soybean, and *H. armigera*, respectively. The expression levels of the target genes were quantified using the 2^–ΔΔ*Ct*^ method (Livak and Schmittgen, 2001). Unless otherwise indicated, the wild-type untreated samples were set to a relative value of 1. All the primers used for qRT-PCR are listed in Supplementary Table 1.

### Subcellular Localization of GmUGT-YFP Fusion Proteins

The full-length CDS of *GmUGT* was amplified with two primers, *GmUGT*–CDS-F/R (Supplementary Table 1). The PCR product was subcloned into the pEarlyGate 101 vector (Deguchi et al., 2020) under the control of the CaMV35S promoter using the ClonExpress II One Step Cloning Kit (Vazyme, Nanjing, China). This yielded a pEarlyGate101-*GmUGT*-YFP construct, which was transformed into *Agrobacterium* strain GV31011 by electroporation. The *Nicotiana benthamiana* plants were grown in a plant growth chamber at 26°C under a 16 h/8 h (light/dark) regimen until they reached heights of approximately 10–20 cm. The plantlets were then infiltrated with *Agrobacterium* strain GV3101 containing pEarlyGate101-GmUGT-YFP. Infiltration was performed as described by Bai et al. (2013). The YFP alone was used as the control, and FIBRILLARIN2-mCherry (FIB2-mCherry) was used as a nucleolar marker (Missbach et al., 2013). The agroinfiltrated leaves were photographed 72 h after infiltration. The fluorescing fusion protein was visualized by confocal microscopy with excitation at 520 nm for YFP and 570–620 nm for FIB2-mCherry.

### Metabolomics Analysis

For the metabolomics analysis, samples were isolated from the WT and the *ko-3* leaves; these leaves included unattacked and attacked samples by *H. armigera* for 36 h and were taken from the same place on the plants. Nine fully expanded leaves from nine independent plants were pooled as one composite biological replicate. Three biological replicates were performed. Biological samples were extracted, and the sample extracts were analyzed using a ultra-performance liquid chromatography with electrospray ionization tandem mass spectrometry (UPLC–ESI–MS/MS) system as described previously (Chen et al., 2013). Significantly regulated metabolites between groups were determined by variable importance in projection (VIP) ≥ 1 and absolute log2 fold change (log2FC) ≥ 1. The VIP values were extracted from the Orthogonal Partial Least Squares Discrimination Analysis (OPLS-DA) results, which contained scores and permutation plots generated using the R package, MetaboAnalystR 3.0^3^. The data were log-transformed (log2) and mean-centered before OPLS-DA. Identified metabolites were annotated using the KEGG Compound database^4^, and annotated metabolites were then mapped to the Kyoto Encyclopedia of Genes and Genomes (KEGG) Pathway database^5^. Pathways with significantly regulated metabolites were mapped and then fed into metabolite set enrichment analysis (MSEA). Their significance was determined by the *p* values of the hypergeometric tests (Thevenot et al., 2015; Zhang X. L. et al., 2017).

### RNA-Seq and Bioinformatics Analysis

Six fully expanded leaves from three independent WT and *ko-3* plants after *H. armigera* larval attack and unattack were pooled as a single biological replicate. The three biological replicates were used for total RNA extraction followed by RNA-seq analysis. The RNA-seq experiment and preliminary data analysis were carried out by the Beijing Biomarker Technology Corporation (Biomarker, Beijing, China). The total RNA was extracted, and sequencing libraries were generated as previously described (Kou et al., 2021). Clean reads were mapped to the soybean reference genome (*G. max* Wm82.a2.v1) downloaded from Phytozome using SOAP aligner/SOAP2. Differentially expressed genes (DEGs) relative to control samples were identified with the absolute value of | log2 FC| ≥ 1 and false discovery rate (FDR) <0.5 as the threshold. The KEGG pathway enrichment of the DEGs was generated using R using enrichment factors, Q-values, and the number of enriched genes in this pathway (Chen et al., 2020).

### Statistical Analysis

All data were collected and analyzed using Microsoft Office Excel 2010. All data analyses were performed by Student’s *t*-test to compare the means of two samples (or treatments) to determine significant differences. Unless otherwise specified, experiments were conducted in triplicates. The values are given as mean ± SD. The consistency between RNA-seq and qRT-PCR analyses was evaluated by the Pearson correlation method based on log2FC. Pearson correlation analyses were performed using GraphPad Prism 8.0 software (GraphPad Software, San Diego, CA, United States).

## Results

### CRISPR/Cas9-Mediated Mutagenesis of *GmUGT* in Soybean Enhanced Resistance to *Helicoverpa armigera* and *Spodoptera litura*

**FIGURE 1:**
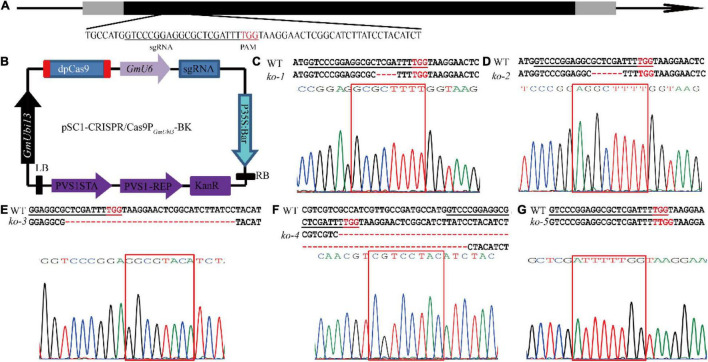
Target site in the *GmUGT* gene and results obtained from the mutagenesis of *GmUGT* through CRISPR/Cas9 technology. **(A)** Schematic figure of the target site in *GmUGT*. Nucleotides in red represent the protospacer adjacent motif (PAM). Nucleotides underlined indicate the target site. **(B)** Schematic figure of the binary vector designed for mutagenesis of the *GmUGT* gene using CRISPR/Cas9 technology. The pSC1-CRISPR/Cas9-P*_*GmUbi*3_*-BK was derived from VK005-04-soU6-2-*GmUbi3*. The sgRNA with a targeting site was directed by the *GmU6* promoter. **(C–G)** Detailed sequence of the target site in T2 generations of the separated lines. ‘–’ signs indicate the number of deleted nucleotides. The WT represents Tianlong No. 1 wild type sequence.

To better understand the *GmUGT* gene and its function in soybean resistance against leaf-chewing insects, *Agrobacterium*-mediated transformation was used to introduce the CRISPR/Cas9 expression vector into the soybean cultivar Tianlong No. 1 (wild type, WT). This generated mutants of the *Glyma.07g110300* gene with a guide RNA targeting *GmUGT* (Figures 1A,B). We obtained four T0 transgenic lines with insertions of the *Bar* gene (Supplementary Figures 1A,B). Several T1 generations were sequenced to identify the CRISPR/Cas9-induced mutant lines, and five edited mutants were identified (Figures 1C–G and Supplementary Figure 1C). The *ko-1*, *ko-2*, *ko-3*, and *ko-4* mutants had 4-bp, 6-bp, 33-bp, and 65-bp deletions, respectively, while *ko-5* had a single-bp insertion in the target site, resulting in altered amino acid sequence or frameshift mutations in the protein encoded by *GmUGT* (Figures 1C–G and Supplementary Figure 2A). Due to the possibility of off-targeting, potential off-target sites predicted by the website CCtop^6^ were also sequenced to detect all cleavages in the mutants (Supplementary Figure 2B). We did not observe a significant difference in plant growth and development between the five homozygous mutants and the WT plants (Supplementary Figure 1C). The ‘transgene-clean’ homozygous *ko-3* and *ko-5* mutants were used for further analysis (Supplementary Figures 1D–F).

**FIGURE 2:**
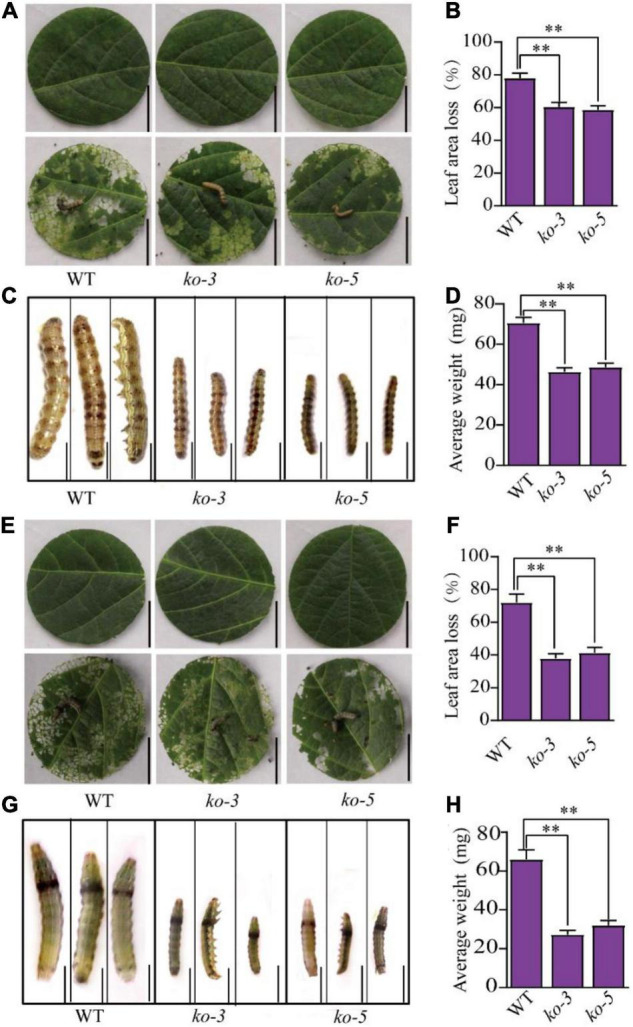
The CRISPR/Cas9-mediated mutagenesis of *GmUGT* enhanced resistance to *H. armigera* and *S. litura* in soybean. **(A)** The phenotype of detached leaves of *ko-3*, *ko-5*, and WT plants attacked by *H. armigera* for 3 days (bar = 2 cm). **(B)** Percentage of leaf area loss in *ko-3*, *ko-5*, and WT plants attacked by *H. armigera* for 3 days (*n* = 50 biological repeats). **(C)** The phenotype of *H. armigera* larvae fed detached leaves of *ko-3*, *ko-5*, and WT plants for 7 days (bar = 0.2 cm). **(D)** The average weight of *H. armigera* larvae that were fed detached leaves of *ko-3*, *ko-5*, and WT plants for 7 days (*n* = 50 larvae). **(E)** The phenotype of detached leaves of *ko-3*, *ko-5*, and WT that was attacked by *S. litura* for 3 days (bar = 2 cm). **(F)** Percentage of leaf area loss in *ko-3*, *ko-5*, and WT plants attacked by *S. litura* for 3 days (*n* = 50 biological repeats). **(G)** The phenotype of *S. litura* larvae that were fed detached leaves of *ko-3*, *ko-5*, and WT plants for 7 days (bar = 0.2 cm). **(H)** The average weight of *S. litura* larvae that were fed detached leaves of *ko-3*, *ko-5*, and WT for 7 days (*n* = 50 larvae). Data shown are means and standard deviations. Statistically significant differences are marked with asterisks (^**^p < 0.01; Student’s *t*-test).

To test the resistance of the *GmUGT* mutants to leaf-chewing insects, we fed the larvae of *H. armigera* and *S. litura* detached leaves of the *ko-3* and *ko-5* mutants and the WT plants. Figure 2 shows that both the *ko-3* and *ko-5* mutants exhibited resistance to *H. armigera* and *S. litura*. The leaf area loss in the *ko-3* and *ko-5* mutants attacked by *H. armigera* was significantly less than that in the WT plants (Figures 2A,B). The average weight of *H. armigera* fed with the leaves of *ko-3* and *ko-5* mutants was also significantly less than that fed with leaves of the WT plants (Figures 2C,D). Similar results were observed when the larvae of *S. litura* were fed detached leaves of the *ko-3* and *ko-5* mutants and the WT plants (Figures 2E–H). These results demonstrated that the CRISPR/Cas9-mediated targeted mutagenesis of *GmUGT* increased resistance against *H. armigera* and *S. litura* in soybeans.

### Soybean Varieties Overexpressing *GmUGT* Were More Sensitive to *H. armigera* and *S. litura*

**FIGURE 3:**
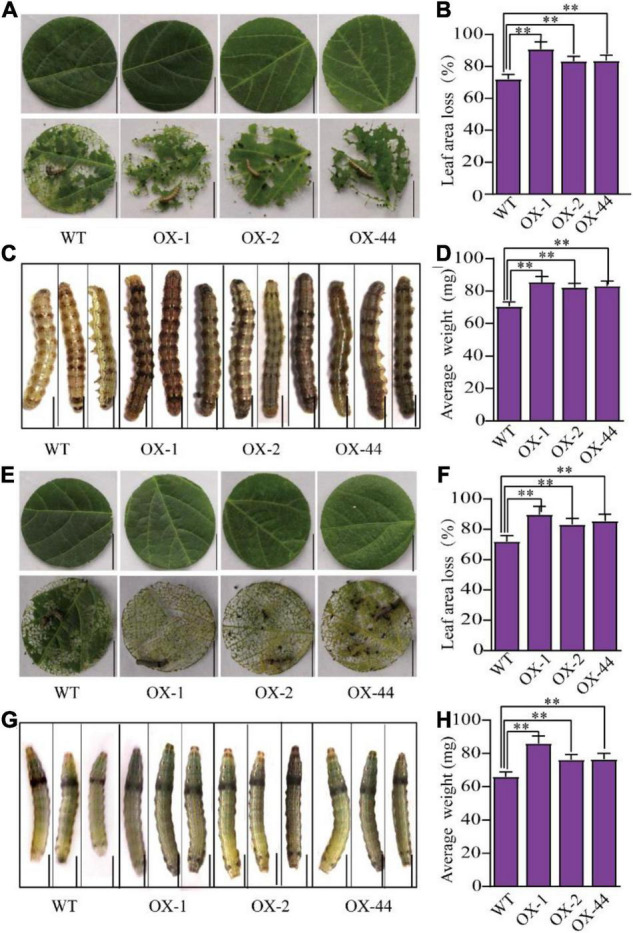
*GmUGT*-OX transgenic soybean plants were susceptible to *H. armigera* and *S. litura*. **(A)** The phenotype of detached leaves of three *GmUGT-*OX transgenic plants (OX-1, OX-2, and OX-44) and WT plants attacked by *H. armigera* for 3 days (bar = 2 cm). **(B)** Percentage of leaf area loss in OX-1, OX-2, OX-44, and WT plants attacked by *H. armigera* for 3 days (*n* = 50 biological repeats). **(C)** The phenotype of *H. armigera* larvae that were fed detached leaves of OX-1, OX-2, OX-44, and WT plants for 7 days (bar = 0.2 cm). **(D)** The average weight of *H. armigera* larvae that were fed detached leaves of OX-1, OX-2, OX-44, and WT plants for 7 days (*n* = 50 larvae). **(E)** The phenotype of detached leaves of OX-1, OX-2, OX-44, and WT plants attacked by *S. litura* for 3 days (bar = 2 cm). **(F)** Percentage of leaf area loss in OX-1, OX-2, OX-44, and WT plants attacked by *S. litura* for 3 days (*n* = 50 biological repeats). **(G)** The phenotype of *S. litura* larvae that were fed detached leaves of OX-1, OX-2, OX-44, and WT plants for 7 days (bar = 0.2 cm). **(H)** The average weight of *S. litura* larvae that were fed detached leaves of OX-1, OX-2, OX-44, and WT plants for 7 days (*n* = 50 larvae). Data shown are means and standard deviations (*n* = 50). Statistically significant differences are marked with asterisks (^**^p < 0.01; Student’s *t*-test).

Because the *GmUGT* mutants (*ko-3* and *ko-5*) showed enhanced resistance to leaf-chewing insects, we wondered whether the overexpression of *GmUGT* would be more sensitive to *H. armigera* and *S. litura.* Four transgenic lines that overexpressed the *GmUGT* gene were generated (OX-1, OX-2, OX32, and OX-44) (Supplementary Figure 3); three of these (OX-1, OX-2, and OX-44) were used to evaluate resistance against *H. armigera* and *S. litura*. As expected, all three *GmUGT*-overexpressing lines were more sensitive to *H. armigera* and *S. litura* in soybean (Figure 3). The loss in leaf area in *GmUGT*-overexpressing lines attacked by *H. armigera* and *S. litura* was more significant than in the WT plants. Furthermore, the average weights of larvae fed *GmUGT*-overexpressing leaves were higher than those that were fed WT leaves (Figure 3). These results suggest that the *GmUGT* gene compromises the resistance of soybeans to leaf-chewing insects.

### Characterization of the *GmUGT* Gene in Soybeans

**FIGURE 4:**
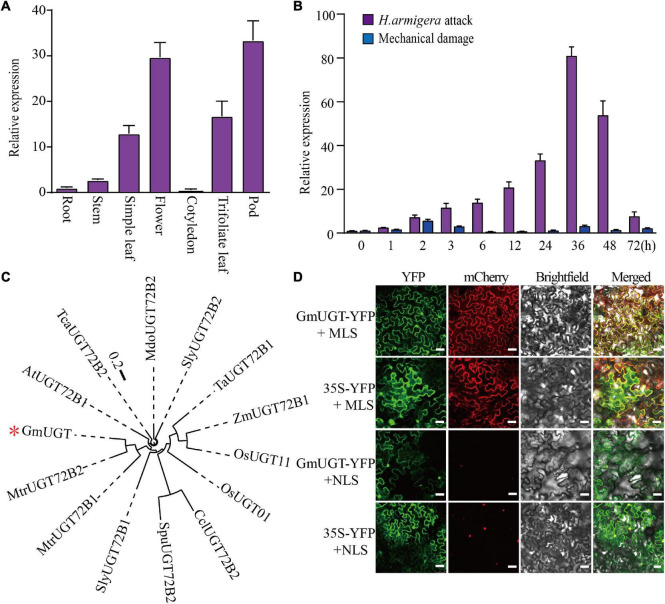
Gene expression analysis, phylogenetic relationship, and localization assay of the *GmUGT* gene. **(A)** Expression of *GmUGT* in roots, stems, simple leaves, flowers, cotyledons, trifoliate leaves, and pods (*n* = 3 biological repeats). **(B)** Expression patterns of *GmUGT* in leaves after attack by *H*. *armigera* and mechanical damage (*n* = 3 biological repeats). **(C)** Phylogenetic analysis of GmUGT and other UGT proteins from diverse species. The scale bar indicates 0.2 substitutions per site. The accession numbers of these proteins in the GenBank database are as follows: GmUGT (Glyma.07g110300); OsUGT01 (LOC_Os01g43280); OsUGT11 (LOC_Os11g38650); AtATUGT72B1 (AT4G01070); MdoUGT72B2 (MDP0000875654); SlyUGT72B2 (Solyc01g095620.2); SlyATUGT72B1 (Solyc04g080010.2); MtrUGT72B1 (Medtr8g006260); MtrUGT72B2 (Medtr1g019510); ZmATUGT72B1 (Zm00008a014945_T01); TaATUGT72B1 (Traes_6AS_43AF9291); TcaUGT72B2 (Thecc1EG005182t1); SpuUGT72B2 (SapurV1A.00130600); and CclUGT72B2 (Ciclev1000172B2). **(D)** Subcellular localization of YFP-GmUGT fusion proteins in the tabacum leaf cells. The fluorescence patterns of GmUGT-YFP and YFP (negative control) were visualized with a high-resolution laser confocal microscope. The extreme left panel shows YFP fluorescence, the middle panel shows mCherry and the bright field, and the right panel represents an overlay of the two images, bar = 75 μm. mCherry, FIBRILLARIN2-mCherry nucleolar marker; MLS, membrane localization signal; NLS, nuclear localization signal.

The tissue expression pattern showed that the *GmUGT* gene was ubiquitously expressed in all tissues. The expression levels in the flower, simple leaf, trifoliate leaves, and pod were higher than those in other tissues (Figure 4A). Leaves are the favorite food of *H. armigera* and *S. litura* larvae. Additionally, Aljbory and Chen (2018) reported that insect attacks can stimulate plant resistance by mechanical damage and/or the application of an elicitor. Thus, we examined the expression patterns of the *GmUGT* gene in the leaves of WT plants after exposure to *H. armigera* attacks and mechanical damage at different times. The transcription of *GmUGT* was induced by *H. armigera* 1 h after the attack, followed by a gradual increase for 36 h and eventually a decrease after 48 h; contrarily, the expression levels of *GmUGT* remained unchanged after mechanical damage (Figure 4B). These results indicate that *GmUGT* is expressed in response to the attack of leaf-chewing insects rather than mechanical damage alone in soybean. Phylogenetic analysis showed that the GmUGT protein shared high sequence homology with ATUGT72B1 in Arabidopsis (Figure 4C), which catalyzes the glucose conjugation of monolignols (Lin et al., 2016). Previous works have reported that genes belonging to the UGT family function as glycosyltransferase in the cytoplasm (Bowles et al., 2005; Sun et al., 2013; Smehilova et al., 2016). To assess the subcellular localization of GmUGT protein, a plasmid carrying *GmUGT* fused with a yellow fluorescent protein (YFP) gene driven by the CaMV 35S promoter was injected into tobacco leaves. The diffused fluorescence of the fusion protein was observed in the cytosol, accumulating along the cell membrane and in the nuclei (Figure 4D), proving GmUGT to be a cytosolic enzyme.

### *GmUGT* Negatively Regulates Flavonoids Content

**FIGURE 5:**
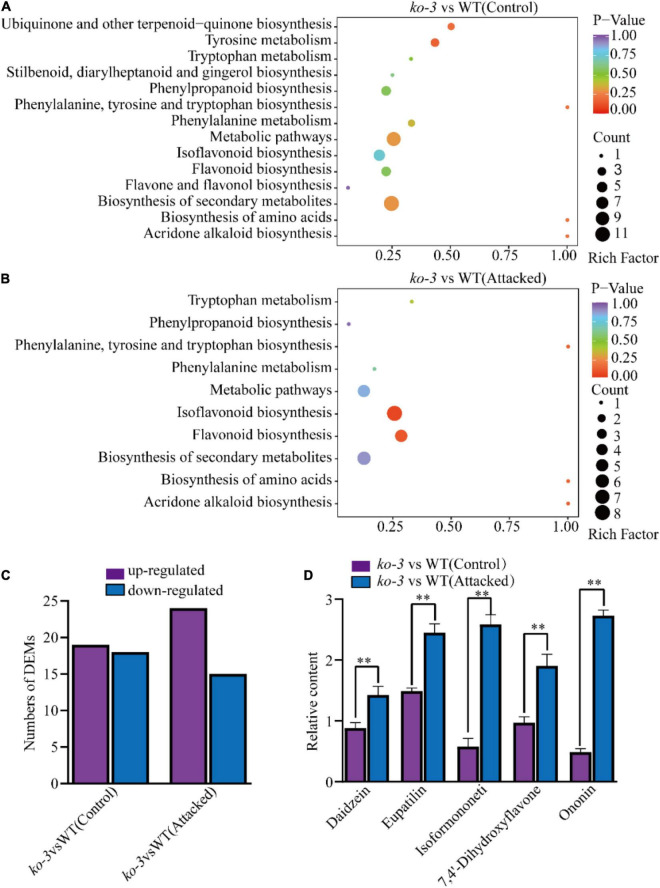
Analysis of different metabolites in *ko-3* versus WT unattacked and attacked by *H. armigera* at 36 h. KEGG pathway enrichment of different metabolites in *ko-3* versus WT plants unattacked *by H. armigera.* **(A)**, and attacked by *H. armigera* at 36 h **(B)**. Each dot represents a KEGG pathway. The Y-axis indicates the KEGG pathway, and the X-axis indicates the rich factor (indicating the degree of differential metabolite enrichment in each pathway; the larger the rich factor is, the greater the differential metabolite enrichment). The dot size and color indicate the number and p value of different metabolites in the pathway, respectively, and a larger dot means more different metabolites. **(C)** The number of different metabolites of flavonoids among *ko-3* versus WT unattacked and attacked by *H. armigera* at 36 h. **(D)** Relative content of metabolites in *ko-3* unattacked and attacked by *H. armigera* at 36 h. The data shown are the means and standard deviations (*n* = 3 biological repeats). Statistically significant differences are marked with asterisks (^**^p < 0.01; Student’s *t-*test).

Because the *Arabidopsis ugt72b1* mutant previously displayed an aggravated cell wall lignification and an enhanced flavonoid content (Lin et al., 2016), we investigated whether *GmUGT*, the homolog of *ATUGT72B1*, plays a similar role in soybeans. First, we evaluated the lignin in the cell walls of the leaves of the *ko-3* and WT plants to find out if they had similar cell wall lignin (Supplementary Figure 4A). To determine the flavonoid content, metabolomics analysis was performed on the *ko-3* and WT plants 36 h after attack or unattack by *H. armigera;* this duration was chosen considering that *GmUGT* gene expression reached its peak after 36 h of attack by *H. armigera* (Figure 4B). Differential metabolite analysis showed that 70 metabolites in unattacked *ko-3* plants mapped to 14 KEGG pathways, while 56 metabolites in the attacked *ko-3* mapped to 10 KEGG pathways (Supplementary Tables 2, 3 and Figures 5A,B). The *GmUGT* encodes a UDP-7-*O*-glucosyltransferase, which catalyzes the final step of flavonoid biosynthesis to form their glycoside derivatives. Loss of function of *GmUGT* resulted in increased daidzein and formononetin contents, while genistein content was reduced due to the redirection of metabolic flux to other products (Supplementary Figures 4B–D). Among these KEGG pathways, isoflavonoid and flavonoid biosynthesis were significantly enriched in the *ko-3* plants attacked by *H. armigera* after 36 h (Figures 5A,B). Different flavonoid metabolites were upregulated in the *ko-3* mutants 36 h after attack by *H. armigera* (Figure 5C). These were related to insect resistance, including 7,4-dihydroxyflavone, eupatilin, isoformononetin, ononin, and daidzein (Figure 5D). Furthermore, we tested the expression levels of 12 immunity-related genes in *H. armigera* larvae that were fed *ko-3* plant leaves for 7 days to observe that the expression of most of these genes was significantly decreased (Supplementary Figures 4E–O). These results indicate that *GmUGT* mutagenesis affects the flavonoid content in soybean attacked by *H. armigera*.

### Differently Expressed Genes in *GmUGT* Mutants and Wild Type Plants

**FIGURE 6:**
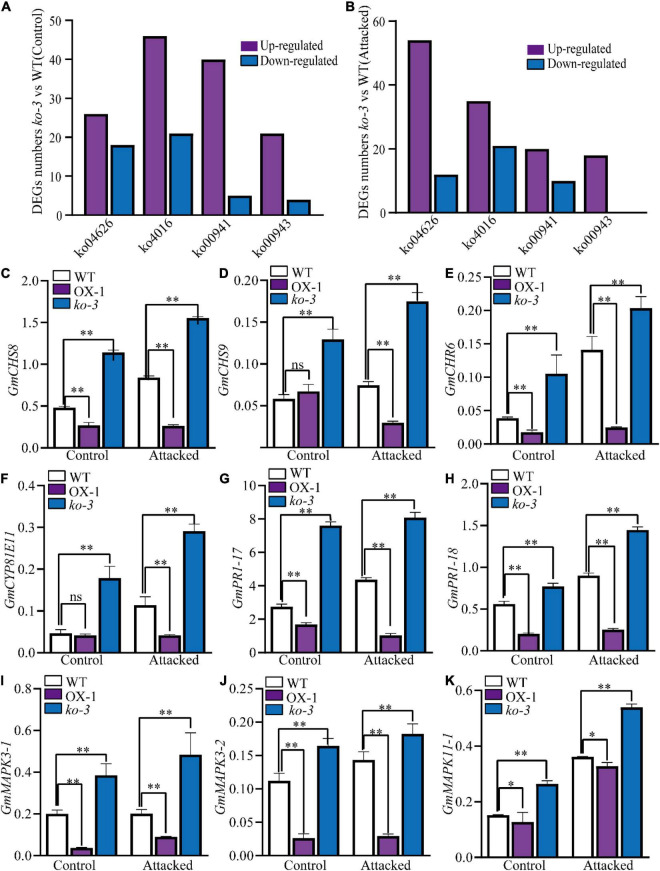
Analysis of differentially expressed genes (DEGs) in *ko-3* versus WT plants unattacked and attacked by *H. armigera* at 36 h. The numbers of DEGs in *ko-3* versus WT plants unattacked by *H. armigera* **(A)** and attacked by *H. armigera* at 36 h **(B)**. **(C–K)** Validation of the expression levels of nine DEGs by qRT-PCR. Relative expression levels were normalized to the expression level of *GmSKIP16*. The data shown are the means and standard deviations (*n* = 3 biological repeats). Statistically significant differences are marked with asterisks (ns *p* > 0.05, 0.01< *p < 0.05, ^**^p < 0.01; Student’s *t-*test; ns, non-significant).

A transcriptome assay was conducted on leaves from the WT, *ko-3* plants that were attacked for 36 h by *H. armigera*, and an unattacked control to identify genes related to resistance against *H. armigera*. The most enriched DEGs in *ko-3* according to the KEGG pathways were associated with isoflavonoid and flavonoid biosynthesis, defensive genes related to plant–pathogen interaction, and mitogen-activated protein kinase (MAPK) signaling pathway (Figures 6A,B, Supplementary Figure 5, and Supplementary Tables 4, 5). The qRT-PCR analysis was employed to investigate the expression of genes involved in flavonoid biosynthesis, including *G. max CHALCONES SYNTHASE* (*GmCHS*), *G. max Chalcone reductase* (*GmCHR*), and *G. max Cytochrome P450 monooxygenase* (*GmCYP81E11*). All three genes were upregulated in *ko-3* as compared to in the WT plants attacked by *H. armigera* (Figures 6C–F). Additionally, genes associated with the defense response were assayed, including *G. max MITOGEN-ACTIVATED PROTEIN KINASE* (*GmMAPK)* and *G. max PATHOGENESIS RELATED1* (*PR1*). These genes were also upregulated in *ko-3* as compared to in the WT plant attacked by *H. armigera* (Figures 6G–K). The consistency between RNA-Seq and qRT-PCR analysis was evaluated by Pearson’s correlation (*R*) analysis based on log2FC revealing an *R* = 0.82 and *p* < 0.0001, which proved the reliability of the RNA-Seq data (Supplementary Figure 5C). These data indicated that the knockout of *GmUGT* enhanced the resistance of soybean against leaf-chewing insects by altering genes related to the flavonoid pathway and defense responses.

### Complementation of Resistance to Leaf-Chewing Insects of *Arabidopsis ugt72b1* Mutant With *GmUGT*

**FIGURE 7:**
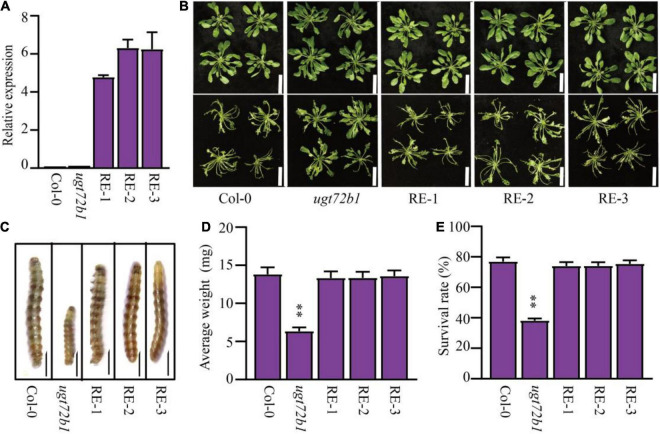
Complementation of resistance to *H*. *armigera* of *Arabidopsis atugt72b1* mutant with *GmUGT*. **(A)** Confirmation of the expression of *GmUGT* in transgenic *Arabidopsis atugt72b1* mutants. Gene expression levels were normalized with Col-0 (AtUGT72B1 expression level) as 1. *AtACTIN2* was used as the internal reference control. The data shown are the means and standard deviations (*n* = 3 biological repeats). **(B)** Phenotype of Col-0, *atugt72b1* and three transgenic lines (RE-1, RE-2 and RE-3) attacked by *H*. *armigera* for 3 days (bar = 2 cm). **(C)** Phenotype of *H. armigera* larvae fed Col-0, *atugt72b1* and three transgenic lines for 7 days (bar = 0.1 cm). **(D)** Average weight of *H*. *armigera* larvae fed Col-0, *atugt72b1* and three transgenic lines for 7 days. Data shown are the means and standard deviation (*n* = 60 larval). **(E)** The survival rate of *H*. *armigera* larvae fed Col-0, *atugt72b1* and three transgenic lines for 7 days (*n* = 3 biological replicates, and 100 *H. armigera* larvae in each replicate). Statistically significant differences are marked with asterisks (ns *p* > 0.05, ^**^p < 0.01; Student’s *t*-test; ns, non-significant).

Because the GmUGT protein shared high homology with the ATUGT72B1 protein (Figure 4C), we hypothesized that the loss-of-function in the *atugt72b1* mutant would show increased resistance to leaf-chewing insects. The larval feeding assay on the *atugt72b1* mutants and the WT plants was performed using the larvae of *H. armigera*. As expected, the *atugt72b1* mutant showed higher resistance to *H*. *armigera* than the WT plants (Supplementary Figure 6). To further validate the role of *GmUGT* in imparting resistance to *H*. *armigera*, we introduced the *GmUGT* gene driven by the *ATUGT72B1* promoter into the *atugt72b1* mutant and obtained three transgenic lines (RE-1, RE-2, and RE-3) with elevated expression of *GmUGT* in *Arabidopsis* (Figure 7A). All three transgenic lines showed a substantial reduction in resistance to *H*. *armigera* (Figure 7), indicating that the UGT gene family is consistently involved in imparting resistance to *H*. *armigera*.

## Discussion

Defoliator insects affect soybean yield and quality, including *H. zea*, *H. armigera*, and *S. litura* (Otuka et al., 2020; Haile et al., 2021). Soybean varieties have been screened for native resistance to such defoliators (Kim et al., 2013, 2015; Liu et al., 2016; Haile et al., 2021). However, breeding such varieties can be labor-intensive and time-consuming (Ortega et al., 2016). Transgenic breeding is a crucial breakthrough in crops breeding for insect resistance. Nevertheless, only six commercialized soybean cultivars with various insect-resistance genes have been identified (Kumar et al., 2020). Among these soybean cultivars, genetically modified cultivars expressing one or more *Bacillus thuringiensis* (Bt) proteins have been widely deployed for pest management (Kumar et al., 2020). This trait provides effective control management for defoliators, including *H. armigera*. Similar to the transgenic crops randomly integrating foreign DNA into plant genomes; the durability, environmental safety, and potential adverse health effects of the Bt transgenic soybeans have always been controversial, limiting the transgenic breeding (Gao, 2021). Recently, CRISPR/Cas9-based genome editing has become popular as it allows the generation of crop varieties similar to non-transgenic crops without the addition of any foreign DNA to the genome (Bao et al., 2019). Here, we presented a strategy to increase the resistance against leaf-chewing insects in soybean through CRISPR/Cas9-mediated targeted mutagenesis of the *UGT* genes through the flavonoid biosynthesis pathway, which can help to accelerate the breeding process for insect resistance and minimize the great concern to human health.

Ortega et al. (2017) reported that *GmUGT* is a functional SNP marker that exhibits activity against *H. zea*, but information about the *GmUGT* gene and its role in imparting resistance to *H. armigera* remains limited. Here, we first characterized the *GmUGT* gene in soybean and observed that it could respond to defoliator attacks rather than mechanical damage (Figure 4B). We observed that the *GmUGT* gene possesses a conserved plant secondary product, the glycosyl-transferase-box (PSPG) motif in its C-terminus (Supplementary Figure 7). The PSPG motif is involved in the recognition and binding of sugar donors (Masada et al., 2007). Additionally, the UGT protein can transfer glycosyl moieties from UDP-sugars to flavonoids during flavonoid biosynthesis (Witte et al., 2009). In soybeans, Yin et al. (2017) identified 212 *UGT* genes and reported that several of them were potentially involved in flavonoid glycosylation. Here, we found that the CRISPR/Cas9-mediated mutagenesis of *GmUGT* led the transgenic plant to exhibit an enhanced resistance to *H. armigera* and *S. litura* (Figure 2). The *Arabidopsis ugt72b1* mutant showed aggravated cell wall lignification and an enhanced flavonoid content (Lin et al., 2016). Both cell wall lignification and flavonoids contribute to resistance against leaf-chewing insects. Cell wall lignification is the first physical barrier against leaf-chewing insects (War et al., 2012). As a secondary metabolite, flavonoids protect plants against leaf-chewing insects by influencing insect behavior, growth, and development (Belete, 2018). Recently, Yang et al. (2021) reported that CRISPR/Cas9-mediated targeted mutagenesis of the *OsUGT707A3* gene in rice (*Oryza sativa*) led to more accumulation of naringenin, lowered naringenin-7-*O*-b-D-glucoside and apigenin-7-*O*-b-D-glucoside, and increased the resistance to insects, such as *S. litura*. Thus, we investigated whether the enhanced resistance against leaf-chewing insects due to GmUGT mutations was attributable to aggravated lignification of cell walls and alteration of flavonoids. Our results indicated that the leaf cell wall lignification and GmUGT mutations were not significantly related (Supplementary Figure 4A).

Metabolomics analysis revealed that loss of function of *GmUGT* altered flavonoids in soybean leaves (Figure 5). The expression levels of immunity-related genes in *H. armigera* larvae, including pattern recognition receptors (PRRs) and antimicrobial peptides (AMPs) (Wang et al., 2010), decreased significantly in *H. armigera* larvae that were fed with *ko-3* plant leaves for 7 days, indicating that long-term feeding with *ko-3* plant leaves may destroy the immune systems of larvae (Supplementary Figures 4E–O). Therefore, the enhanced resistance of soybean due to *GmUGT* mutations against leaf-chewing insects may be dependent on altered flavonoids.

Transcriptome assay and qRT-PCR analysis showed that the genes involved in flavonoid biosynthesis were upregulated in *ko-3* plants after the *H*. *armigera* attack. Previous studies have shown that the genes encoding the CHS, CHR, and CYP81E11 enzymes play a critical role in flavonoid biosynthesis (Dhaubhadel et al., 2007; Zhang X. B. et al., 2017; Mameda et al., 2018; Pi et al., 2019; Zuk et al., 2019). One of the earliest signaling events after leaf-chewing insect attack is the activation of mitogen-activated protein kinases (MAPKs), which are essential to activate the transcription of defense-related genes and accumulate associated metabolites in plants. For example, *MAPKs* can upregulate the defense gene, *GmPR-1*, when plants are attacked by insects (Hettenhausen et al., 2015; Giacometti et al., 2016; Breen et al., 2017; Fabricio and Venancio, 2021). Additionally, flavonoids that function as signal molecules may directly interfere with MAPK activities and profoundly affect signaling systems (Agati et al., 2013). Consistent with this, we found out that several genes that were involved in the MAPK pathway, along with the *GmPR-1* genes, were upregulated in *ko-3* compared with the WT plants upon attack by *H*. *armigera* (Figure 6). These results indicate that the *GmUGT* gene plays an essential role in controlling resistance to leaf-chewing insects through the flavonoid biosynthesis pathway in soybeans.

Interestingly, the *Arabidopsis ugt72b1* mutant also exhibited increased resistance to *H*. *armigera* (Supplementary Figure 6), while GmUGT shared high homology with AtUGT72B1 (Figure 4C). Additionally, our results showed that the ectopic expression of the *GmUGT* gene in the *Arabidopsis ugt72b1* mutant substantially lessened the resistance of *Arabidopsis ugt72b1* mutant to *H. armigera* (Figure 7). Lin et al. (2016) reported that *ugt72b1* mutant exhibited growth repression after bolting, while the *ugt72b1* mutant phenotype was restored to the WT by expression of *AtUGT72B1*. However, we observed that the *Arabidopsis ugt72b1* mutant was not restored to its WT by expressing *GmUGT* (Supplementary Figure 8). Furthermore, mutations and overexpression of *GmUGT* did not cause any noticeable phenotypic changes (Supplementary Figures 1C, 3A). These results indicate that the two homologous genes, *AtUGT72B1* and *GmUGT*, are functionally differentiated, but they play a conserved role in imparting resistance to leaf-chewing insects.

## Conclusion

The CRISPR/Cas9-mediated mutagenesis of *GmUGT* increased soybean resistance to *H. armigera* and *S. litura* by altering flavonoid content and by changing the expression of genes related to flavonoid biosynthesis and defense response. The *atugt72b1* mutant of *Arabidopsis* also exhibited an increased resistance to *H. armiger*, while the ectopic expression of the *GmUGT* gene in this mutant substantially reduced the resistance against *H. armigera*. Our findings highlight an effective strategy for increasing resistance to leaf-chewing insects in soybean through CRISPR/Cas9-mediated targeted mutagenesis of the *UGT* genes, which may also work for other crops.

## Data Availability Statement

The datasets presented in this study can be found in online repositories. The names of the repository/repositories and accession number(s) can be found below: https://www.ncbi.nlm.nih.gov/, SAMN21448187, SAMN21448188, SAMN21448189, SAMN21448190, SAMN21448191, SAMN 21448192, SAMN21448193, SAMN21448194, SAMN21448195, SAMN21448196, and SAMN21448197.

## Author Contributions

YZ, WG, DC, XL, and YJ designed the experiments. YZ, WG, LC, XS, HY, YF, WO, SM, HC, SC, QH, YH, DQ, ZS, ZY, SY, CZ, and XZ performed the experiments and analyzed the data. YZ, WG, DC, and XL wrote the manuscript. All authors contributed to the article and approved the submitted version.

## Conflict of Interest

The authors declare that the research was conducted in the absence of any commercial or financial relationships that could be construed as a potential conflict of interest.

## Publisher’s Note

All claims expressed in this article are solely those of the authors and do not necessarily represent those of their affiliated organizations, or those of the publisher, the editors and the reviewers. Any product that may be evaluated in this article, or claim that may be made by its manufacturer, is not guaranteed or endorsed by the publisher.

## Footnotes

1
http://cbi.hzau.edu.cn/crispr/

2
https://cctop.cos.uni-heidelberg.de:8043

3
https://github.com/xia-lab/MetaboAnalystR

4
http://www.kegg.jp/kegg/compound/

5
http://www.kegg.jp/kegg/pathway.html

6
https://cctop.cos.uni-heidelberg.de:8043
