# Supplementary material for: CRISPR/Cas9-Mediated Targeted Mutagenesis of GmUGT Enhanced Soybean Resistance Against Leaf-Chewing Insects Through Flavonoids Biosynthesis
Source: Front Plant Sci. 2022 Feb 22;13:802716. doi: 10.3389/fpls.2022.802716 (PMC8902248; doi:10.3389/fpls.2022.802716)
Supplement: Supplementary file 2 [file Data_Sheet_1.docx]

**Supplemental Materials**

**Supplementary Figure 1** **|** Identification of transgene-clean mutagenesis of *GmUGT* in soybean. **(A)** PCR analysis of transgenic lines with the *Bar* gene in T1 generations of the separated lines. M: 2000bp DNA marker; 2,5,6,8, individual transgenic lines showed Bar positive; 1,3,4,7,9,10, individual mutant lines showed *Bar* negative. **(B)** Detection of the Bar protein by Transgenic Plant Bar Gene Rapid Detection Kit. 2,5,6,8, individual transgenic lines showed Bar positive (the bands at red arrowhead). **(C)** Phenotype of five CRISPR/Cas9-mediated targeted mutagenesis of *GmUGT* (*ko-1*, *ko-2*, *ko-3*, *ko-4* and *ko-5*) and WT plants grown in an artificial climate chamber under 14-h light/10-h dark photoperiod conditions at 26°C for 35 days (bar = 15cm). **(D)** Detection of the Bar protein of T2 generations of *ko-1*, *ko-2*, *ko-3*, *ko-4* and *ko-5* mutants by Transgenic Plant Bar Gene Rapid Detection Kit. The *ko-1*, *ko-2* and *ko-4* mutants showed Bar positive while *ko-3* and *ko-5* mutants showed Bar negative. PCR analysis of transgenic lines with the *Bar* gene (**E**) and *Cas9* gene (**F**) in T2 generations of the separated lines. The *ko-1*, *ko-2* and *ko-4* mutants showed *Bar* and *CAS9* positive while *ko-3* and *ko-5* mutants showed *Bar* and *CAS9* negative. **+:** The pSC1-CRISPR/Cas9-P_GmUbi3_-BK plasmid was selected as positive control. **-:** The Tianlong No. 1 DNA was selected as negative control.

**Supplementary Figure 2** **|** Multiple alignments of the amino-acid sequence of five mutants and off-target detection of *Glyma.09G245300* gene in soybean. **(A)** Multiple alignment of the amino-acid sequence of five CRISPR/Cas9-mediated targeted mutagenesis of *GmUGT* (*ko-1*, *ko-2*, *ko-3*, *ko-4* and *ko-5*) and WT plants. **(B)** Off-target detection of *Glyma.09G245300*. Top, comparison of target sites of *GmUGT* and *Glyma.09G245300* gene. Bottom, detailed sequence of the target site in *Glyma.09G245300* gene in T2 generations of the separated lines. The underlined nucleotides indicated the off-target site. The red box indicated the sequencing peak map of the off-target site of *Glyma.09G245300* gene.

**Supplementary Figure 3** **|** Identification of overexpressing *GmUGT* transgenic lines in soybean. **(A)** Phenotype of four transgenic lines overexpressing *GmUGT* gene (OX-1, OX-2, OX32 and OX-44) and WT plants grown in an artificial climate chamber under 14-h light/10-h dark photoperiod conditions at 26°C for 35 days (bar = 15cm). **(B)** PCR analysis of transgenic lines using *GmUGT*-PB2GW7specific primers. M: 2000bp DNA marker; 1-3: OX-1; 4-6: OX-2; 7-9: OX-32; 10-12: OX-44; **(C)** Detection of the Bar protein of T2 generations of OX-1, OX-2, OX32 and OX-44 by Transgenic Plant *Bar* Gene Rapid Detection Kit. 1-3: OX-1; 4-6: OX-2; 7-9: OX-32; 10-12: OX-44; Four transgenic lines all showed Bar positive (the bands at red arrowhead). **(D)** Relative expression of *GmUGT* in T2 generations of OX-1, OX-2, OX-32, and OX-44. *GmSKIP16* was used as the internal reference control. Gene expression levels were normalized with WT as 1. Data shown are means and standard deviations (*n* = 3 biological repeats).

**Supplementary Figure 4 |** Lignin content of cell wall and expression patterns of *H. armigera* immunity-related genes in *H. armigera*. **(A)** Lignin content (ug/mg) of cell wall in wild-type plants leaves and *ko-3* plants leaves. **(B-D)** Relative content of metabolites in *ko-3* plants. Daidzin: daidzein-7-O-glucoside; Ononin: formononetin-7-O-glucoside; Genistin: Genistein-7-O-Glucoside. (**E-P**) Expression patterns of *H. armigera* immunity-related genes in *H. armigera* fed with wild-type plants leaves (white column) and *ko-3* plant leaves (purple column). *H. armigera* actin (*HaActin*) was used as an internal standard to normalize the templates. Data shown are means and standard deviations (*n* = 3 biological repeats). Statistically significant difference between WT and *ko-3* is marked with asterisk(s) (0.01< * *p* < 0.05, ** *p* < 0.01; Student’s *t*-test).

**Supplementary Figure 5 |** Top 20 KEGG pathway enrichment analysis of the differentially expressed genes (DEGs). KEGG pathway enrichment analysis of DEGs in *ko-3* versus WT unattacked by *H.armigera* (Control) **(A**) and attacked by *H.armigera* at 36 hours **(B)**. Each dot represents a KEGG pathway. The Y-axis indicates the KEGG pathway, the X-axis indicates the GeneRatio (the ratio of annotated genes in the item to the numbers of all DEGs). The dot size and color indicate the number and p-Value of DEGs in the pathway respectively, the numbers near dots mean the counts of DEGs. (**C**) Correlation of qRT-PCR results with RNA-seq on 9 genes. R represents the Pearson correlation coefficients of qRT-PCR expression data versus RNA-seq data. R= 0. 82, *p* <0.0001.

**Supplementary Figure 6** **|** *Arabidopsis* *atugt72b1* mutant plants enhanced resistance to *H. armigera*. **(A)** Top, phenotype of Col-0 and *ugt72b1*mutant before attack; Bottom, phenotype of Col-0 and *ugt72b*1mutant attacked by *H. armigera* for 3 days (bar=2cm). **(B)** Average weight of *H. armigera* fed with detached leaves of Col-0 and *ugt72b1* mutant for 7 days. Data shown are means and standard deviations (n = 35 larval). Statistically significant difference is marked with asterisks (***p* < 0.01; Student’s *t*-test). **(C)** Phenotype of *H. armigera* larva fed with Col-0 and *ugt72b1* mutant for 7, respectively (bar = 0.1cm).

**Supplementary Figure 7** **|** PSPG-box consensus sequence of *Vitis vinifera,* *Medicago truncatula, Arabidopsis thaliana* and *Glycine max* uridine diphosphate glycosyltransferase

**Supplementary Figure 8** **|** Phenotype of Col-0, *atugt72b1* and three transgenic lines (RE-1, RE-2, and RE-3) after about 7 weeks of growth (bar=5cm).

**Supplementary Table 1 |** Primers used in plasmid construction, PCR and qRT-PCR analysis.

**Supplementary Table 2 |** Different metabolites in *ko-3* versus WT plants unattacked by *H.armigera* with the |log2 (Fold Change)| ≥1 and Variable Importance in Projection（VIP）≥1.

**Supplementary Table 3 |** Different metabolites in *ko-3* versus WT plants attacked by *H.armigera* at 36 hours with the |log2 (Fold Change)| ≥1 and Variable Importance in Projection（VIP）≥1.

**Supplementary Table 4 |** Differentially expressed genes in *ko-3* versus WT plants unattacked by *H.armigera* with the |log2 (Fold Change)| ≥1 and false discovery rate (FDR) ≤ 0.05.

**Supplementary Table 5 |** Differentially expressed genes in *ko-3* versus WT plants attacked by *H.armigera* at 36 hours with the |log2 (Fold Change)| ≥1 and false discovery rate (FDR) ≤ 0.05.
