# Supplementary figures and images for: CRISPR/Cas9-Mediated Targeted Mutagenesis of GmUGT Enhanced Soybean Resistance Against Leaf-Chewing Insects Through Flavonoids Biosynthesis
Source: Front Plant Sci. 2022 Feb 22;13:802716. doi: 10.3389/fpls.2022.802716 (PMC8902248; doi:10.3389/fpls.2022.802716)

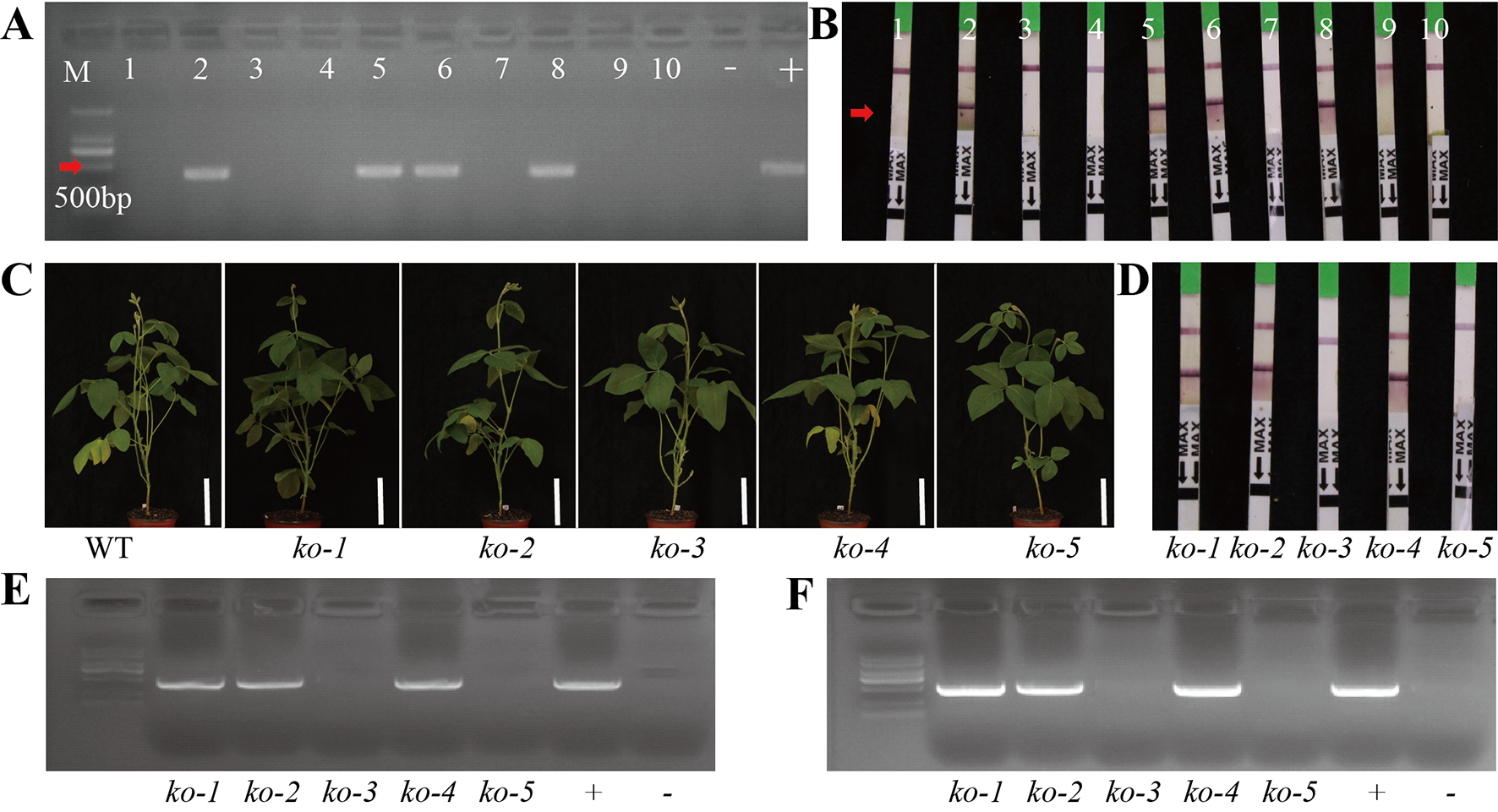

Supplement: Supplementary file 3 [file Image_1.TIF]

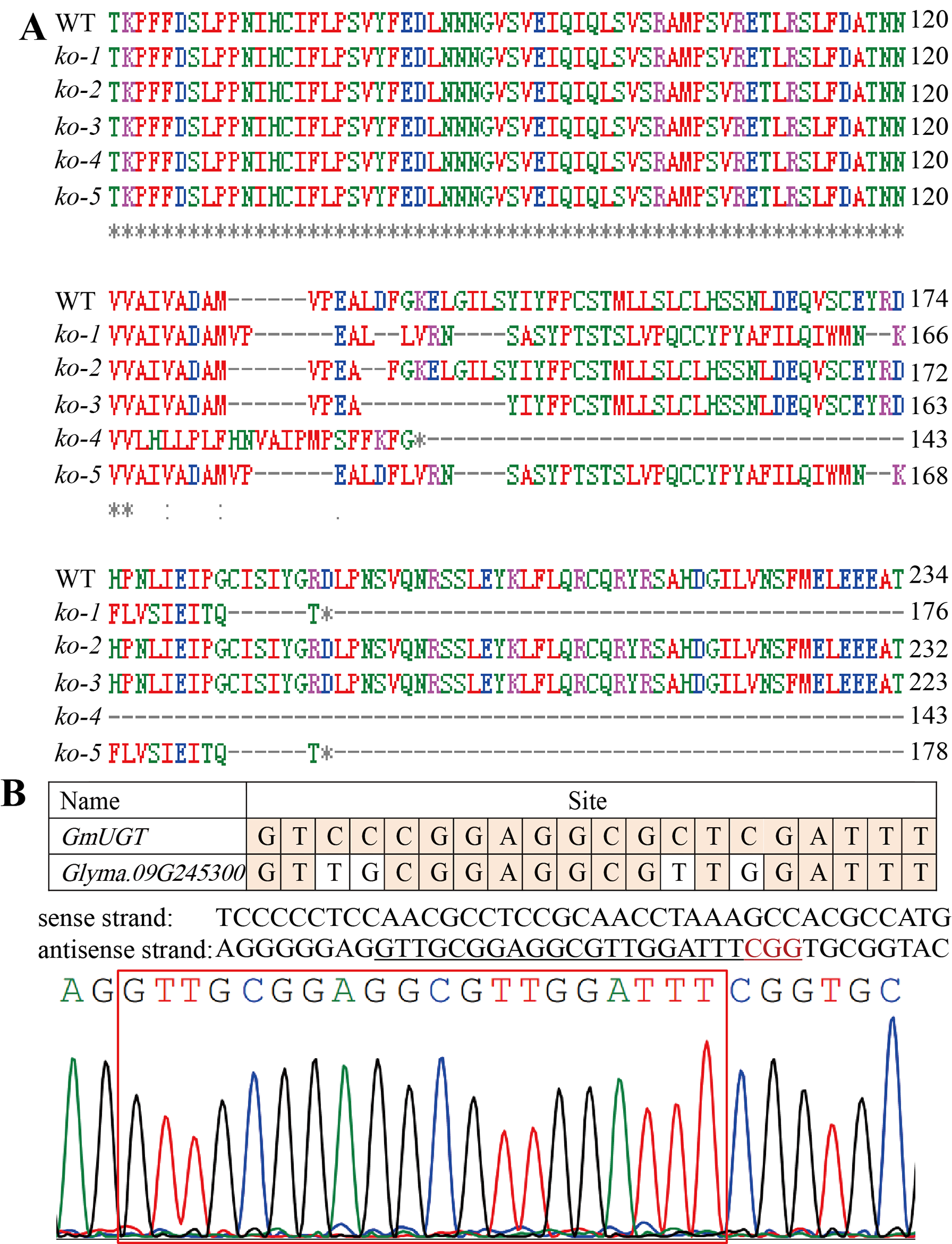

Supplement: Supplementary file 4 [file Image_2.TIF]

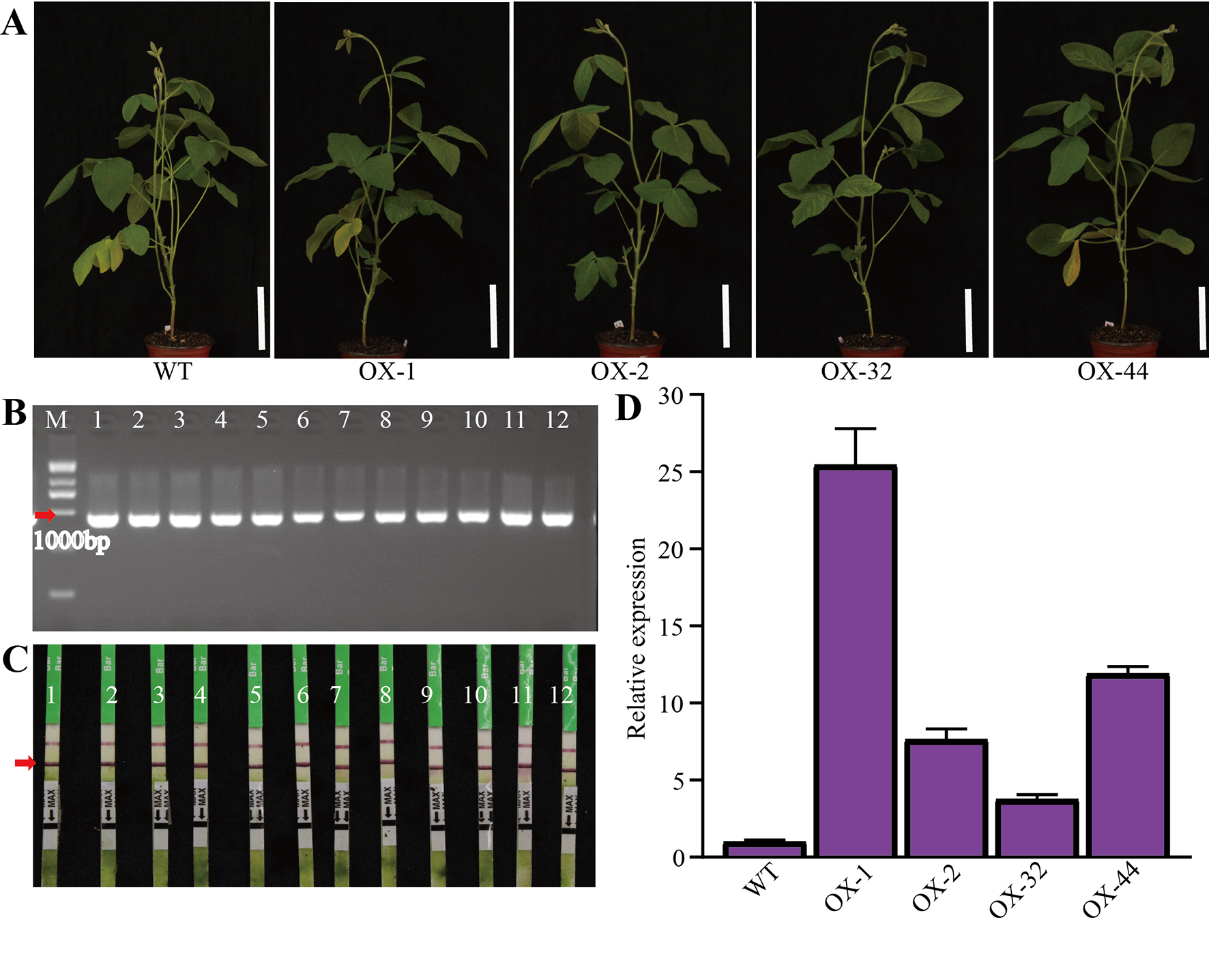

Supplement: Supplementary file 5 [file Image_3.TIF]

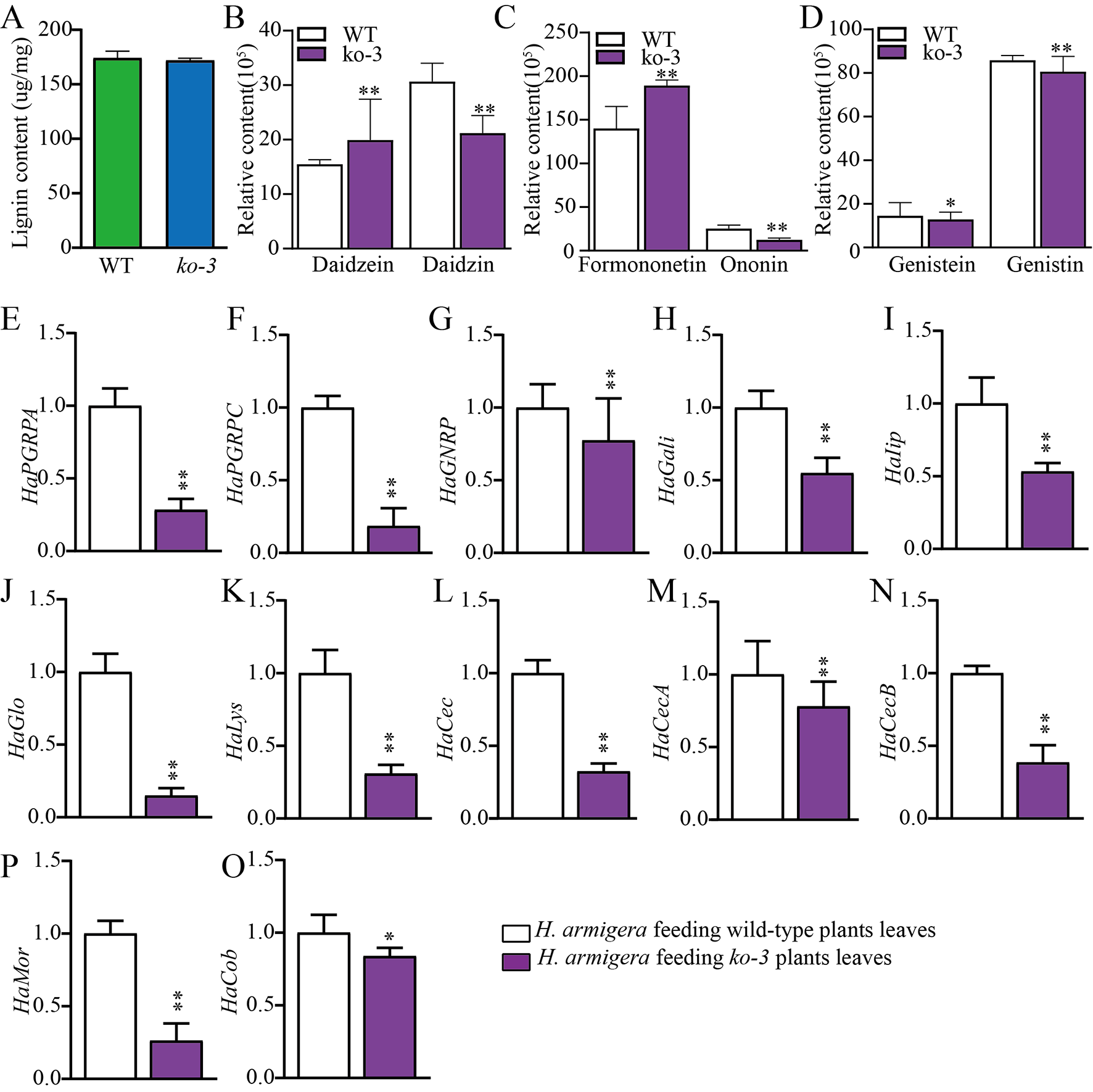

Supplement: Supplementary file 6 [file Image_4.TIF]

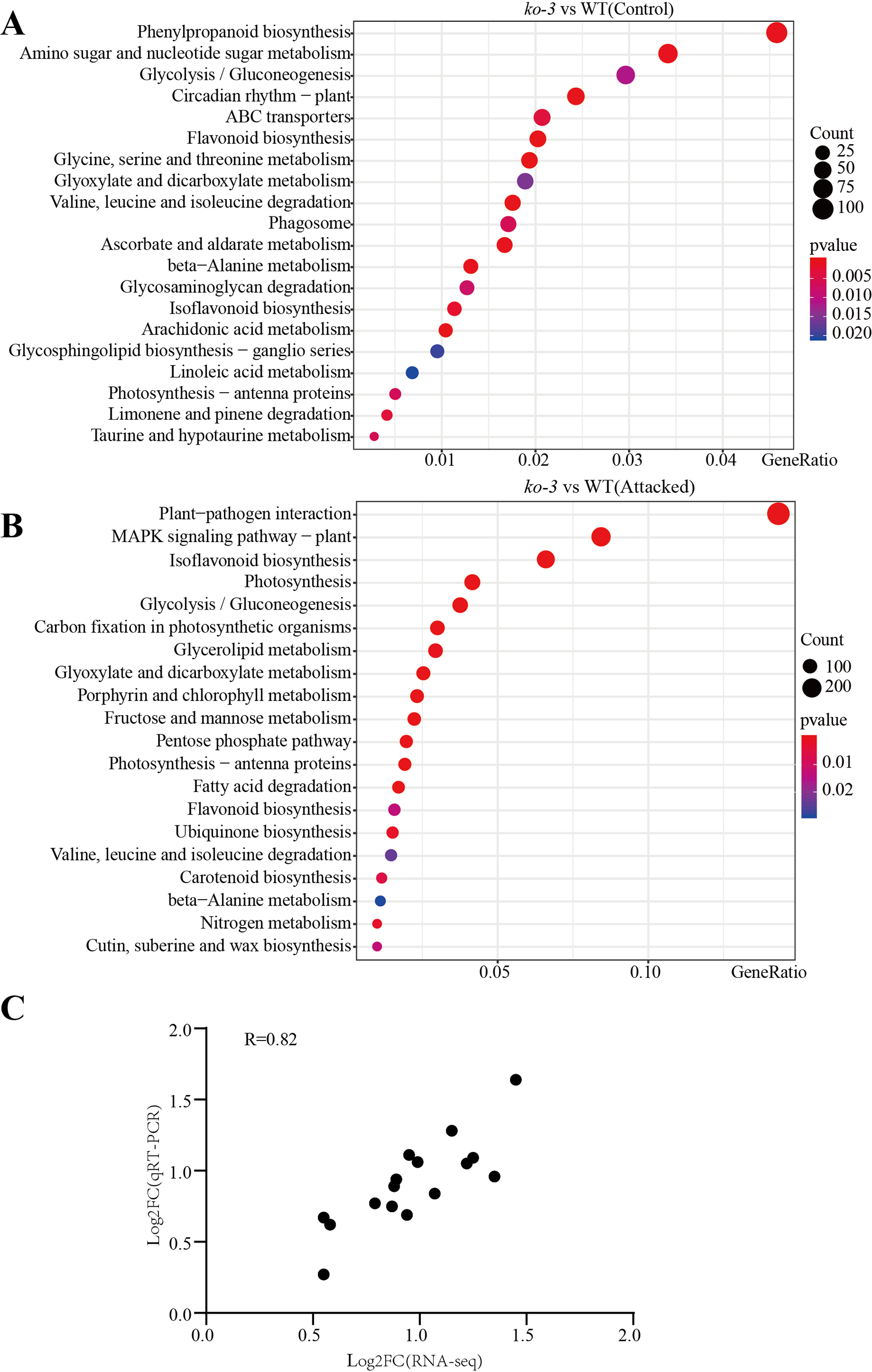

Supplement: Supplementary file 7 [file Image_5.TIF]

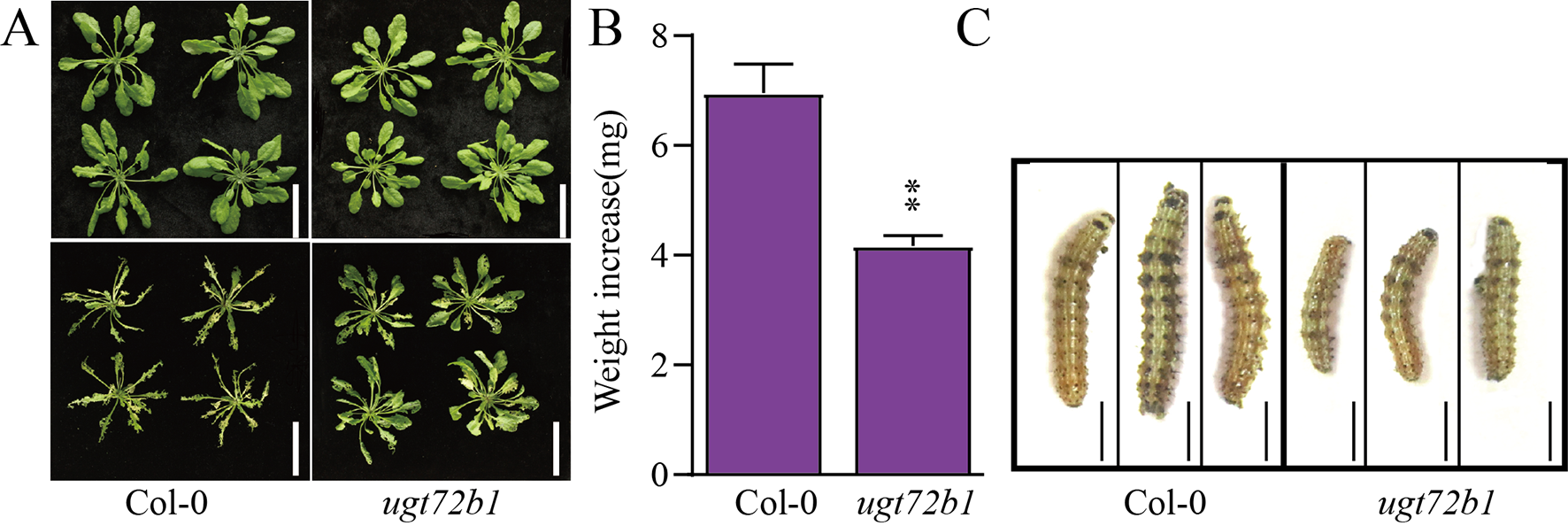

Supplement: Supplementary file 8 [file Image_6.TIF]

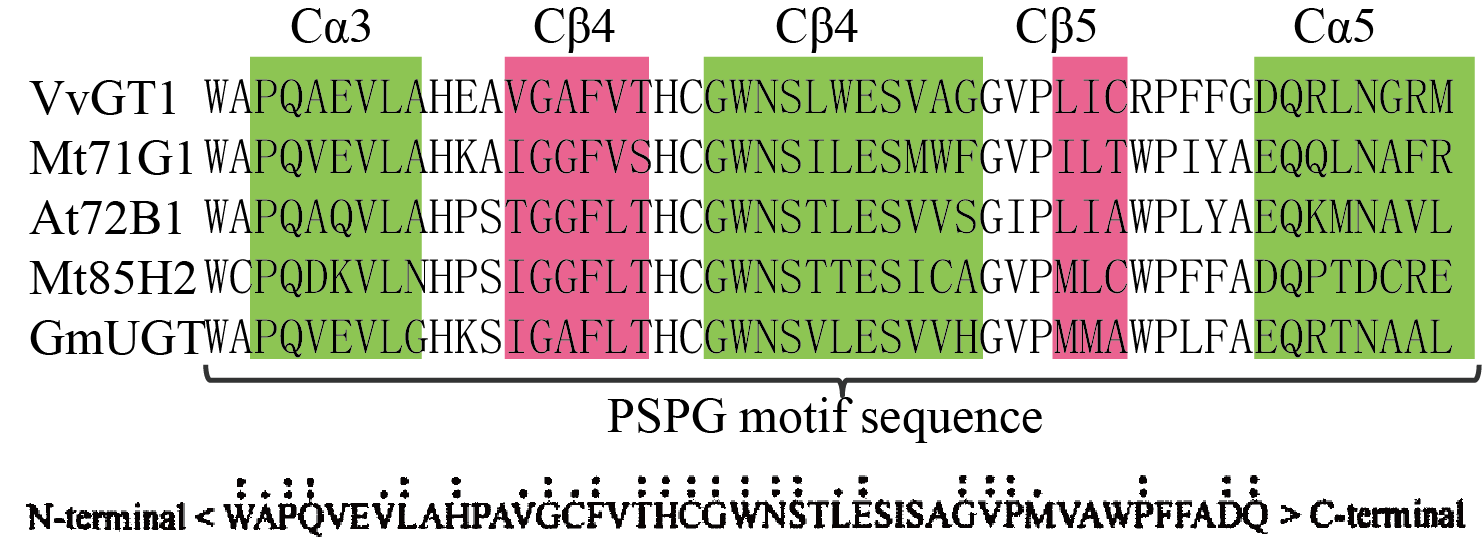

Supplement: Supplementary file 9 [file Image_7.TIF]

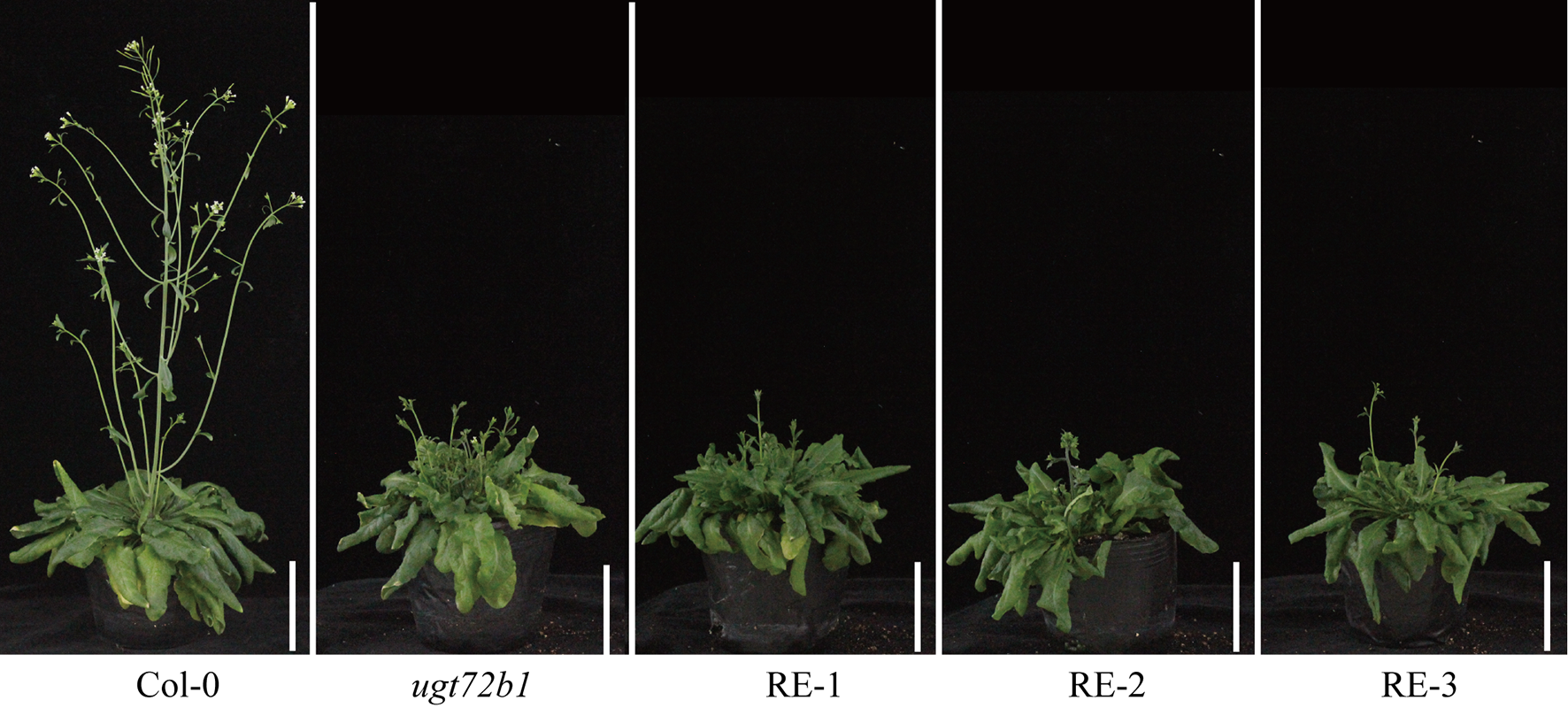

Supplement: Supplementary file 10 [file Image_8.TIF]
